# A Case of an Atypical Presentation of Spontaneous Esophageal Rupture

**DOI:** 10.7759/cureus.57578

**Published:** 2024-04-04

**Authors:** Rashed Aldoseri, Mai Nasser, Mohamed Alshehabi

**Affiliations:** 1 Otolaryngology - Head and Neck Surgery, Royal Medical Services, Riffa, BHR

**Keywords:** radiology, ent, case report, boerhaave syndrome, spontaneous esophageal rupture

## Abstract

Spontaneous esophageal rupture is an uncommon medical phenomenon that involves a sudden increase in intraesophageal pressure with negative intrathoracic pressure. Here, a 21-year-old female with no history of medical illness was admitted to our accident and emergency department with a one-day history of sudden retrosternal chest pain with other symptoms. There was no foreign body ingestion, vomiting, fever, cough, trauma, or recent procedures. Physical examination revealed a soft abdomen with epigastric tenderness and normal respiratory and cardiovascular examinations. The patient underwent a chest X-ray and a computed tomography scan of the neck and chest, which revealed retropharyngeal air extending to the mediastinum with anterior chest surgical emphysema. Oesophago-gastro-duodenoscopy revealed mild gastritis with no evidence of foreign body or esophagus injury. The patient was prescribed paracetamol, pantoprazole, and clindamycin. On follow-up, the patient was doing well with no active complaints. Conservative management of spontaneous esophageal rupture can result in good clinical outcomes with no requirement for additional interventions.

## Introduction

Spontaneous esophageal rupture, also known as Boerhaave syndrome, is an uncommonly seen medical phenomenon that was first described by Dr. Herman Boerhaave in 1724. The pathophysiology is based on a combination of a sudden increase in intraesophageal pressure with negative intrathoracic pressure, such as that commonly seen in patients with forceful vomiting or severe straining [1]. In many cases, the clinical presentation is very non-specific; thus, many patients, more than 50%, are either diagnosed late or misdiagnosed entirely. Clinical diagnosis should not be based on history alone, but a patient presenting with a triad of vomiting, subcutaneous emphysema, and chest pain, known as Mackler’s triad, should increase a physician’s suspicion of an esophageal tear [2]. Diagnosis and management of an esophageal rupture should be prompt; a delay in treatment can give rise to several life-threatening complications, namely, septic shock, mediastinitis, and pleural empyema. Management can be done conservatively, endoscopically, or surgically, and the decision as to which the best approach to take is dependent on a number of different factors [3]. We present a case of a 21-year-old female with no significant medical history who was admitted to our hospital due to a spontaneous esophageal rupture in the absence of any precipitating factors.

## Case presentation

**Figure 1:**
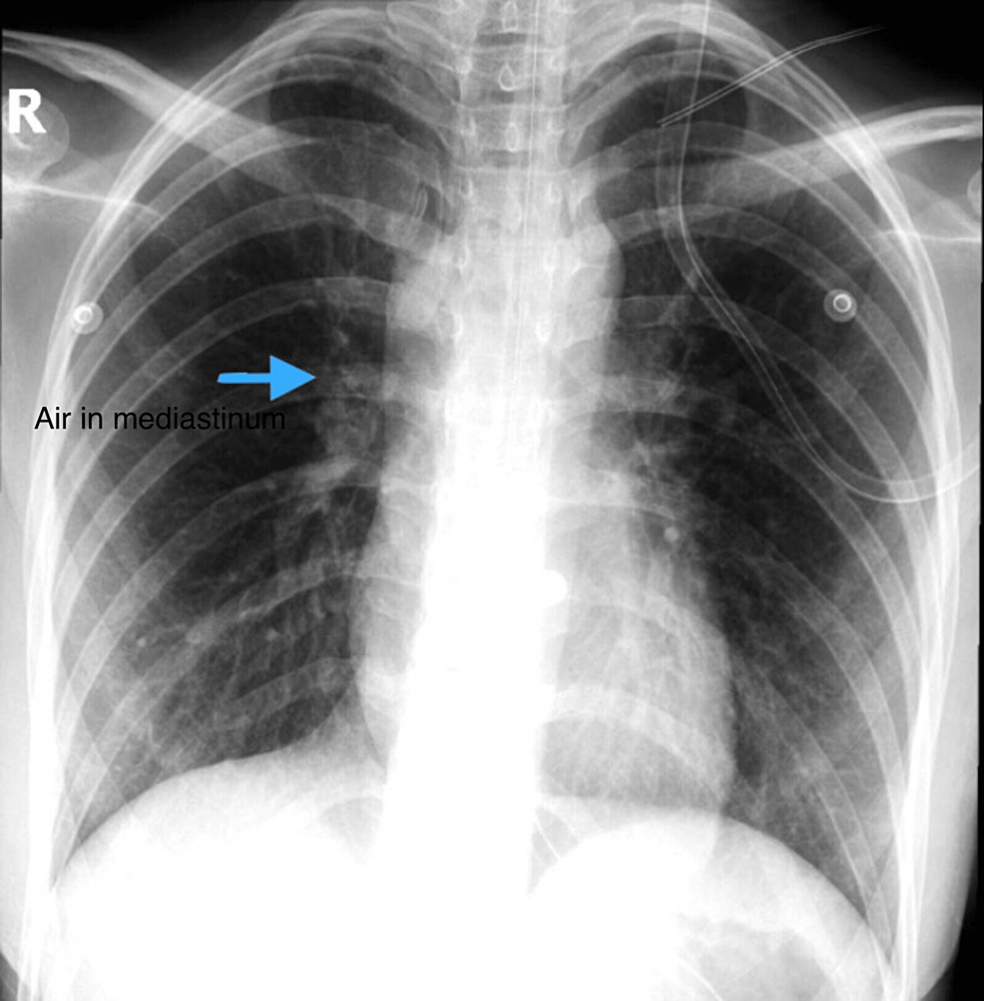
Chest X-ray shows air in the mediastinum.

**Figure 2:**
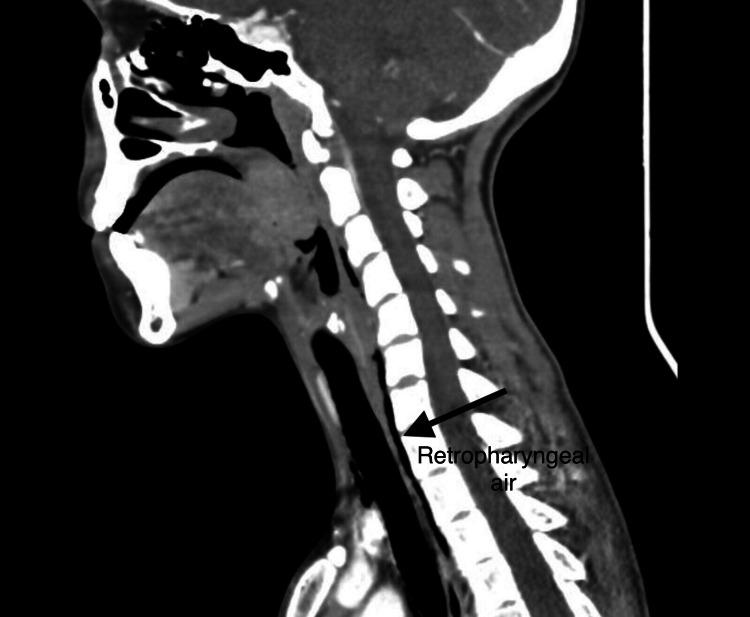
CT scan of neck and chest shows retropharyngeal air.

A 20-year-old female, not known to have any medical illnesses, presented to our accident and emergency department with a one-day history of sudden retrosternal chest pain. The pain was central, pricking in nature, increasing in severity, and radiating to her neck, causing throat pain and difficulty in swallowing. Associated symptoms include a change in her voice recently and shortness of breath, which was exacerbated when lying down flat on the bed. The patient denied any history of foreign body ingestion, vomiting, fever, cough, trauma, and recent procedures. At the time of admission, she was vitally stable with a heart rate of 80 bpm, blood pressure of 108/55 mmHg, respiratory rate of 16 breaths/min, oxygen saturation of 99% on room air, and body temperature of 36.8 °C. The patient was neurologically intact and was conscious, alert, oriented, and in pain. Physical examination revealed a soft abdomen with epigastric region tenderness and normal bowel sounds. On respiratory examination, bilateral equal air entry with no added sounds was noted, and no signs of subcutaneous air and tracheal deviation were present. Cardiovascular examination displayed normal heart sounds with no murmurs. The remaining of her physical examination was unremarkable. A series of investigations were done to help diagnose spontaneous esophageal rupture. Laboratory blood investigations, such as serum electrolytes, complete blood count, and liver and renal tests, were all within normal ranges. A chest X-ray was done, which indicated the presence of air in the mediastinum, and a neck X-ray showed an accumulation of retropharyngeal air (Figure 1). A computed tomography (CT) scan of the neck and chest revealed retropharyngeal air extending to the mediastinum with anterior chest surgical emphysema and no definite foreign body (Figure 2). Lastly, an oesophageal-gastro-duodenoscopy (OGD) revealed mild gastritis with no evidence of foreign body or injury to the esophagus. 

**Figure 3:**
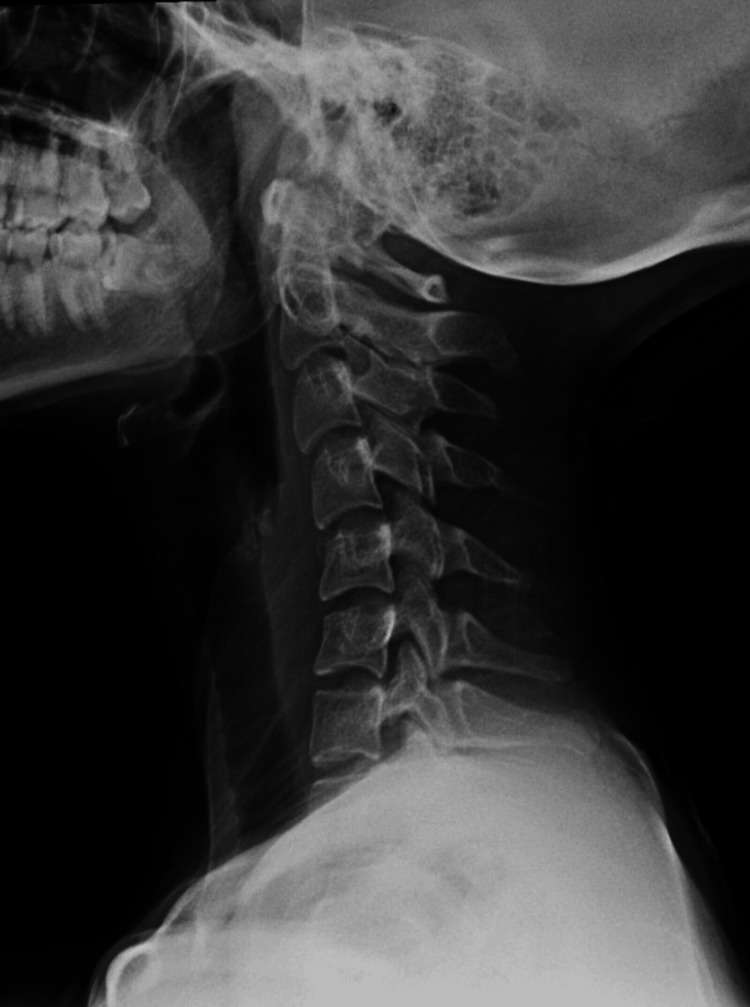
Neck X-ray shows no presence of soft tissue air or any fluid collection.

**Figure 4:**
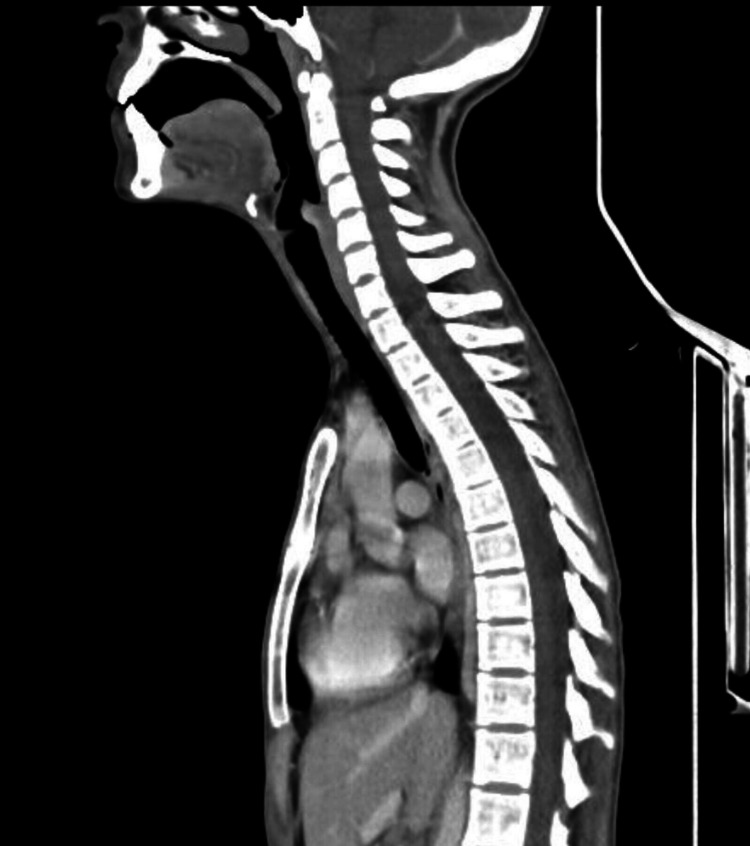
CT scan of neck and chest shows no evidence of retropharyngeal air or mediastinal emphysema.

A 16 Fr nasogastric tube was inserted over a guidewire under direct visualization. The patient was kept on NPO status (nothing by mouth) with close monitoring of her vitals and symptoms and was started on IV fluids, pantoprazole, dexamethasone, and clindamycin. On the next day, she was vitally stable, and all her symptoms subsided, including her pain and shortness of breath. The Gastrografin study followed by the Barium study was done, and both were insignificant. As a result of these investigations, the nasogastric tube was removed, and the patient was started on a clear fluid diet, which progressed to a soft food diet. The rest of the patient's stay in the hospital was unremarkable. After monitoring the improvement of the patient's condition, she was deemed fit to be discharged on paracetamol, pantoprazole, and clindamycin. One week post-discharge, she presented to the ENT clinic for a follow-up. The patient was doing well with no active complaints. She denied having any pain or any difficulty in breathing. An X-ray of the neck and a contrast-enhanced CT scan of the neck and chest revealed that the retropharyngeal air and mediastinal emphysema were completely resolved, with no evidence of soft tissue air nor any fluid collection (Figures 3, 4). These radiological features correlate with a marked improvement in the patient's clinical condition. The patient was given instructions to avoid efforts for a short period of time and required no further evaluation and follow-up.

## Discussion

Esophageal perforation is a serious gastrointestinal emergency that, if left untreated, can lead to complications. A multitude of causes can lead to esophageal perforation, the most common of which is iatrogenic perforation. An upper gastrointestinal endoscopy is the leading cause of iatrogenic perforation of the esophagus. Other non-iatrogenic causes include foreign body-induced perforation, chemical-induced perforation, post-trauma perforation, perforation of the diseased esophagus, and, lastly, spontaneous perforation [1,4]. Spontaneous esophageal rupture is a rare medical condition with an incidence rate of 3.1 per 1,000,000 per year that compromises 15% of all total esophageal perforations [5]. It is caused by a rapid, sudden increase of intraluminal pressure of the esophagus and a decrease in the intrathoracic pressure, and thus repeated vomiting and retching are the most common precipitating factors for this condition [6]. However, 12-50% of patients do not have a history of prior vomiting [7].

Our presented case of spontaneous esophageal tear is unique due to the absence of any precipitating factor. As already aforementioned, a 20-year-old female with no prior medical history presented to our emergency room with non-specific signs and symptoms. A detailed history was taken to identify any probable precipitating factors that could have induced the perforation, such as vomiting, chronic cough, seizure, and straining, all of which were not present. Vital signs, physical examination, and blood investigations were insignificant, and no cause for the patient's clinical picture could be established. However, radiological investigations all indicated findings relating to a possible esophageal rupture. The multiple radiological studies, combined with the unremarkable history, physical examination, and blood investigations, all supported the diagnosis of a spontaneous esophageal rupture with no precipitating factor.

The clinical presentation of the patient depends on two important aspects: the severity of the esophageal perforation and its site. In cervical esophagus perforation, as observed in our case, the most common signs are subcutaneous emphysema (95%) and cervical pain (90%), and in some cases, a fever, dysphagia, or dysphonia may be present [1]. Furthermore, although Mackler’s triad, which consists of chest pain, repeated vomiting, and subcutaneous emphysema, is indicative of esophageal rupture, it only occurs in a minority of cases. Around 50% of all patients present atypically [8]. The non-specificity of the clinical presentation leads to a wide range of differential diagnoses that need to be ruled out before spontaneous esophageal perforation can be diagnosed, some of which include myocardial infarction, aortic dissection, perforated ulcer, acute pancreatitis, spontaneous pneumothorax, and pulmonary disease [9]. When management is delayed, the risk of developing severe complications, such as sepsis, multi-organ failure, respiratory failure, and mediastinitis, increases. The estimated mortality rate ranges between 16% and 24% [6], with the highest mortality rate being noted in those with thoracic esophageal perforation, followed by abdominal perforation and cervical perforation [10]. Radiological investigations have a significant role in the diagnosis of an esophageal tear. An X-ray of patients with cervical esophageal perforation may show gas in the paravertebral fascial planes on the lateral view, tracheal displacement, and subcutaneous emphysema. On the other hand, X-rays of those with thoracic esophageal perforation may demonstrate pneumomediastinum, mediastinal widening, subcutaneous emphysema, and pneumothorax [10]. A CT scan is more sensitive than plain radiography in cases of esophageal perforations due to its ability to effectively illustrate more anatomical details [3]. In addition, a CT scan can help visualize the extension of the disease to adjacent structures and rule out some of the differential diagnoses. Gastrografin and Barium studies provide a good estimate of the location of the perforation and whether the leakage of esophageal content is contained or not [1]. Moreover, endoscopy has a high sensitivity to detect and show the extent of any tear present, but a physician must take caution as it may aggravate the tear and worsen the patient’s clinical status [3]. All of the aforementioned investigations were done in our case to help narrow down our differential diagnosis.

Two different approaches to treatment are established for the management of spontaneous esophageal perforation, conservative and non-conservative. Non-conservative management can be further divided into endoscopic and surgical. Choosing the most appropriate approach to management depends on the stability of the patient, the underlying disease of the esophagus, and the site and size of the perforation [10]. As done in our case, conservative treatment consists of cessation of oral intake and insertion of a nasogastric tube under direct visualization. IV fluids, broad-spectrum antibiotics, and a proton pump inhibitor should be administered, and the patient's clinical status needs to be monitored frequently throughout admission [11]. This approach of management worked well in our case, with no need for further interventions. Endoscopic management is comprised of minimally invasive interventions in the form of endoscopic clipping, endoscopic gluing, and endoprostheses and is indicated in those in which conservative treatment failed. Surgical management consists of a variety of different types of procedures, all of which have been proven to be able to effectively treat and manage a spontaneous esophageal rupture. The established surgical procedures include debridement of necrotic tissue, simple drainage, T-tube placement, suturing of the perforation, reinforcement flaps, and even esophageal resection. Choosing which procedure is best suited to treat the patient is dependent on the site of rupture and should be decided on a case-to-case basis [4].

## Conclusions

Diagnosing an idiopathic spontaneous esophageal rupture requires a high index of suspicion from the physician. Efficiently taking a proper history can help rule out many of the possible etiological factors of this disease, and prompt investigations are key in establishing the diagnosis and avoiding serious complications brought by a delayed or missed diagnosis, which may be fatal. Several radiological imaging modalities are available that can reveal specific findings indicative of spontaneous esophageal rupture and can be used as a guide to monitor a patient's response to treatment. Finally, although the effects of spontaneous esophageal rupture can be quite devastating, conservative management can result in good clinical outcomes with no necessity for further interventions.

## References

[1] Chirica M, Champault A, Dray X, Sulpice L, Munoz-Bongrand N, Sarfati E, Cattan P (2010). Esophageal perforations. J Visc Surg.

[2] Jougon J, Mc Bride T, Delcambre F, Minniti A, Velly JF (2004). Primary esophageal repair for Boerhaave's syndrome whatever the free interval between perforation and treatment. Eur J Cardiothorac Surg.

[3] Spapen J, De Regt J, Nieboer K, Verfaillie G, Honoré PM, Spapen H (2013). Boerhaave's syndrome: still a diagnostic and therapeutic challenge in the 21st century. Case Rep Crit Care.

[4] Wu JT, Mattox KL, Wall MJ Jr (2007). Esophageal perforations: new perspectives and treatment paradigms. J Trauma.

[5] Fleury L, Johnson N, Silberman M (2017). Spontaneous esophageal rupture: a case of back pain. Int J Acad.

[6] Harikrishnan S, Murugesan CS, Karthikeyan R, Manickavasagam K, Singh B (2020). Challenges faced in the management of complicated Boerhaave syndrome: a tertiary care center experience. Pan Afr Med J.

[7] Ringstrom E, Freedman J (2006). Approach to undifferentiated chest pain in the emergency department: a review of recent medical literature and published practice guidelines. Mt Sinai J Med.

[8] Aga Z, Avelino J, Darling GE, Leung JJ (2016). An unusual case of spontaneous esophageal rupture after swallowing a boneless chicken nugget. Case Rep Emerg Med.

[9] Søreide JA, Viste A (2011). Esophageal perforation: diagnostic work-up and clinical decision-making in the first 24 hours. Scand J Trauma Resusc Emerg Med.

[10] Lampridis S, Mitsos S, Hayward M, Lawrence D, Panagiotopoulos N (2020). The insidious presentation and challenging management of esophageal perforation following diagnostic and therapeutic interventions. J Thorac Dis.

[11] Waweru P, Mwaniki D (2015). Conservative management of an iatrogenic esophageal tear in Kenya. Case Rep Surg.

